# The Effect of Semen Cryopreservation Process on Metabolomic Profiles of Turkey Sperm as Assessed by NMR Analysis

**DOI:** 10.3390/biology11050642

**Published:** 2022-04-22

**Authors:** Gianluca Paventi, Michele Di Iorio, Giusy Rusco, Anatoly P. Sobolev, Silvia Cerolini, Emanuele Antenucci, Mattia Spano, Luisa Mannina, Nicolaia Iaffaldano

**Affiliations:** 1 Department of Medicine and Health Sciences “Vincenzo Tiberio”, University of Molise, 86100 Campobasso, Italy; paventi@unimol.it; 2 Department of Agricultural, Environmental and Food Sciences, University of Molise, 86100 Campobasso, Italy; michele.diiorio@unimol.it (M.D.I.); giusy.rusco@unimol.it (G.R.); e.antenucci@studenti.unimol.it (E.A.); 3 Institute for Biological Systems, Magnetic Resonance Laboratory “Segre-Capitani”, CNR, 00015 Monterotondo, Italy; anatoly.sobolev@cnr.it; 4 Department of Veterinary Medicine, University of Milano, 26900 Lodi, Italy; silvia.cerolini@unimi.it; 5 Department of Chemistry and Technology of Drugs, Sapienza University of Rome, 00185 Rome, Italy; mattia.spano@uniroma1.it (M.S.); luisa.mannina@uniroma1.it (L.M.)

**Keywords:** sperm metabolic profile, turkey sperm cryopreservation, avian sperm, sperm NMR analysis

## Abstract

**Simple Summary:**

Semen cryobanking is a valuable tool for preserving the genetic resources of a wide range of species, providing the opportunituy to preserve representative samples and reconstruct the population or diversity. However, in avian species, the freezing–thawing process results in a sharp reduction in sperm quality and consequently fertility. This is mainly due to the lack of knowledge about the molecular basis of the cryopreservation process, especially in more sensitive species such as turkey. Thus, in this study, we took advantage of NMR technology to assess the changes in metabolic profile occurring in turkey sperm cryopreservation, which were correlated with sperm qualitative parameters measured in both fresh and frozen–thawed samples. Hence, the results reported here depict a clearer scenario about the changes in the levels of amino acids, other water-soluble compounds, and lipids resulting from the freezing–thawing process. Moreover, a wide discussion about the possible pathway affected by cryopreservation is provided. Therefore, this study allows us to: *(i)* identify biological markers related to the sperm freezability of male turkey donators; *(ii)* suggest a supplementation of specific metabolites in the diet or in the freezing medium in order to obtain spermatozoa abler to withstand the freezing process.

**Abstract:**

Semen cryopreservation represents the main tool for preservation of biodiversity; however, in avian species, the freezing–thawing process results in a sharp reduction in sperm quality and consequently fertility. Thus, to gain a first insight into the molecular basis of the cryopreservation of turkey sperm, the NMR-assessed metabolite profiles of fresh and frozen–thawed samples were herein investigated and compared with sperm qualitative parameters. Cryopreservation decreased the sperm viability, mobility, and osmotic tolerance of frozen–thawed samples. This decrease in sperm quality was associated with the variation in the levels of some metabolites in both aqueous and lipid sperm extracts, as investigated by NMR analysis. Higher amounts of the amino acids Ala, Ile, Leu, Phe, Tyr, and Val were found in fresh than in frozen–thawed sperm; on the contrary, Gly content increased after cryopreservation. A positive correlation (*p* < 0.01) between the amino acid levels and all qualitative parameters was found, except in the case of Gly, the levels of which were negatively correlated (*p* < 0.01) with sperm quality. Other water-soluble compounds, namely formate, lactate, AMP, creatine, and carnitine, turned out to be present at higher concentrations in fresh sperm, whereas cryopreserved samples showed increased levels of citrate and acetyl-carnitine. Frozen–thawed sperm also showed decreases in cholesterol and polyunsaturated fatty acids, whereas saturated fatty acids were found to be higher in cryopreserved than in fresh sperm. Interestingly, lactate, carnitine (*p* < 0.01), AMP, creatine, cholesterol, and phosphatidylcholine (*p* < 0.05) levels were positively correlated with all sperm quality parameters, whereas citrate (*p* < 0.01), fumarate, acetyl-carnitine, and saturated fatty acids (*p* < 0.05) showed negative correlations. A detailed discussion aimed at explaining these correlations in the sperm cell context is provided, returning a clearer scenario of metabolic changes occurring in turkey sperm cryopreservation.

## 1. Introduction

Conservation of genetic variability in domestic animal species is a task for the sustainable production of human food resources, as well as for land management and, more importantly, the preservation of biodiversity [1,2]. Currently, despite the development of other innovative strategies, such as gonadal tissue allotransplantation [3] and diploid primordial germ cell methodologies [4], semen cryopreservation still remains the most effective method to store reproductive cells for the ex situ management of genetic diversity in birds [5,6,7]. Thus, the growth of this ex situ in vitro strategy, as a support to the in vivo strategy, is becoming more advanced. In this regard, the constitution of cryobanks for genetic resources would offer a crucial link between both strategies, leading to the improvement of conservation programs efficiency [8,9].

However, the main challenge for the creation of a poultry semen cryobank is the achievement of a successful freezing protocol [9]. In relation to this, it is known that the semen cryopreservation process causes a loss of sperm membrane integrity in poultry [2,5,10]. Thus, the improvement of sperm cryosurvival and increased fertility in artificial insemination (AI) with frozen–thawed sperm continues to be the focus in semen cryobanking in avian species. This is even more evident in turkey species, the semen of which has been proven to be more sensitive to cooling and freezing–thawing damages than chicken sperm [11,12,13,14,15,16,17]. Though chicken and turkey sperm share the same morphology (as a filiform shape, a long tail and a condensed nucleus [18]), turkey sperm presents a high cholesterol/phospholipid ratio, resulting in low membrane fluidity and permeability [18]; moreover, it has a low osmotic resistance at hypo-osmotic conditions [11].

For these reasons, over the last few decades, several studies have been performed, in which different factors involved in sperm cryosurvivability were taken into consideration, to identify an efficient freezing procedure for both chicken and turkey semen [14,19,20,21,22,23,24,25]. However, despite the encouraging results obtained so far, there is still a gap of knowledge about the biological bases involved in the cryopreservation process. This limits the development of a freezing procedure that results in fertilization rates closer to those obtained with fresh semen. In addition, more efficient semen cryopreservation, besides ensuring the conservation of genetic resources in a gene bank, could provide practical advantages to the turkey industry, since commercial farms are completely dependent on AI to obtain fertile eggs [14]. The avian sperm membrane contains more polyunsaturated fatty acids (PUFAs) than that of mammals, and it has lower protein content, a lower cholesterol/phospholipid ratio, and greater overall fluidity at physiological temperatures [26,27].

Specific biological and biophysical factors, such as membrane permeability, lipid composition, and membrane fluidity, can affect the ability of poultry spermatozoa to limit damages caused by the cryopreservation procedure [28,29]. In this regard, it was reported that the freezing–thawing procedure for avian spermatozoa induces a membrane rigidifying process that is accompanied by a dramatic and proportional decrease in the cholesterol/phospholipid ratio. Moreover, this effect is different from species to species; thus, it could behave as an indicator of between-species freezability [18,28]. Accordingly, it was also observed that the ratio of lipids in the sperm membrane determines the overall fluidity of the membrane and impacts the ability of sperm to remain viable during the cryopreservation process in chicken [18,30] as well as in mammals [31]. This is consistent with previous findings in turkey showing that lipids are involved in vital aspects of sperm metabolism and functions [32]. 

Besides lipids, other factors contribute to defining membrane fluidity in poultry [33], such as the nature and the level of insertion of the proteins in the membrane lipid bilayer [34]. In addition, amino acids could play a role in avian sperm function as shown for mammals: some amino acids participate in many metabolic processes involved in motility, acrosome reaction, and capacitation of human and other mammalian spermatozoa [35,36]. Amino acids also have antioxidant properties able to protect sperm cells from cold shock [37,38]; consistently, plasma amino acids seem to play a role in chicken sperm cryoresistance [39]. In mammals, it has been demonstrated that amino acids act at the extracellular level and improve sperm motility, acrosome integrity, and fertilizing potential after the freezing–thawing process [40,41,42,43,44]. It has also been reported that L-carnitine is involved in sperm energy metabolism, promoting sperm motility and maturation and the spermatogenic process [45,46]. Thus, its supplementation was proposed to increase both kinetics and morphological characteristics of sperm [47]. On the contrary, less information is known about these aspects in avian species, especially in turkey.

The sperm metabolite profile, which includes lipids, amino acids, and other water-soluble compounds, appears to be the main factor that affects sperm resilience the cryopreservation process, which in turn determines the fertilizing ability of sperm [48]. Even now, there is a complete lack of knowledge about the turkey sperm metabolite profile. The only investigation on metabolite profile of fresh turkey sperm reported in literature was our previous study, in which changes in metabolite levels occurring in male reproductive ageing were measured by using nuclear magnetic resonance (NMR) [49]. Currently, there is still no scientific evidence in the literature about the metabolite profile changes during the semen cryopreservation process. Thus, by detecting and simultaneously quantifying a wide range of metabolites with a high analytical precision, NMR represents a valuable tool for better understanding the biological bases of the turkey semen cryopreservation process.

In this study, deeper insight into the metabolite profile changes occurring in turkey sperm cryopreservation was obtained by assessing via NMR a relevant number of metabolites in both fresh and frozen–thawed samples. The changes in metabolite levels were further correlated to sperm quality variations after thawing and are herein discussed from a metabolic point of view.

## 2. Materials and Methods

### 2.1. Chemicals

The fluorescent dyes SYBR-14 and propidium iodide (PI) used were those provided in the LIVE/DEAD Sperm Viability kit (Invitrogen^TM^ by Thermo Fisher Scientific, Waltham, MA, USA). All the other chemicals used in this study were purchased from Sigma Chemical Co. (St. Louis, MO, USA).

### 2.2. Animals and Semen Treatment

Hybrid Large White turkey males from a private breeding group (Agricola Santo Stefano of Amadori’s group, Canzano, TE, Italy) were used. Animals were housed there when they were 32 weeks old. They were maintained under standard management conditions and photostimulated on a daily basis with a 14L:10D photoperiod. The toms were kept in groups of 8–10 in floor pens. Feed and water were provided ad libitum. Toms were trained for semen collection by abdominal massage two times a week.

Semen was collected from 32 weeks of age males by abdominal massage. Ejaculates were pooled, with each pool originating from a minimum of 9 to a maximum of 12 males, and thoroughly mixed in order to reach at least 4 mL of semen/pool. In total, 5 pools of semen were used in this study.

### 2.3. Cryopreservation Process 

Semen was cryopreserved by the pellet method [14]. In brief, semen samples were diluted (1:4) in Tselutin extender [50]. The diluted semen was cooled at 4 °C for 60 min, and then 8% (*v*/*v*; 0.860 M) of dimethylacetamide (DMA) was added as cryoprotectant. The semen was gently inverted and equilibrated for 5 min at 4 °C. Volumes of 80 µL of semen were plunged drop by drop directly into liquid nitrogen to form spheres of frozen semen (pellets). The pellets were rapidly placed in 2 mL polypropylene cryovials (Cryo.s^TM^; Greiner Bio-One, Monroe, NC, USA) previously cooled by immersion in liquid nitrogen (3–4 pellets/cryovial) and then stored in a liquid nitrogen tank until analysis. After two weeks, the pellets were warmed by immersing the cryovials in a water bath at 75 °C for 12 s.

### 2.4. Semen Quality Evaluation

Mobility, viability and sperm osmotic tolerance were assessed on both freshly diluted and frozen–thawed samples. To this end, each sperm sample was divided in two aliquots: one to be immediately assessed (fresh), and the other to be subjected to freezing–thawing process.

Sperm mobility was evaluated using the Sperm Motility Test (SMT) according to the Accudenz^®^ procedure (Accurate Chemical & Scientific Corp., Westbury, NY, USA) following the procedure described by Iaffaldano et al. [14,49]. This procedure is based on the ability of the spermatozoa with a forward progressive motility to penetrate a 4% Accudenz^®^ layer. Semen was diluted to 1.0 × 10^9^ as previously described [49]. A drop of 60 µL from each sperm suspension was superimposed onto 600 µL of 4% (*w*/*v*) Accudenz^®^ solution in a semimicro polystyrene disposable cuvette. Cuvettes were incubated for 5 min in a 41 °C water bath, and absorbance was measured in a spectrophotometer at 550 nm after 60 s. The sperm motility was expressed by values of optical density (O.D.).

Sperm viability was measured using the Invitrogen^TM^ LIVE/DEAD sperm viability kit according to the procedure set up by Iaffaldano et al [14]. Aliquots of 5 µL semen were diluted in 39 µL of Tselutin diluent containing 1 µL of SYBR-14 (diluted 1:100 into dimethylsulfoxide). Samples were incubated for 10 min at 38 °C. Then, 5 µL of propidium iodide (PI; dissolved 1:100 in PBS) was added, and the samples were further incubated at 38 °C for 5 min. The assessment of viable/nonviable spermatozoa was performed using fluorescence microscopy (blue excitation filter λ = 488 nm; ×100 oil immersion objective; magnification ×400). Viable sperm cells were stained green by SYBR-14, whereas dead cells were stained in red by PI. A minimum of 200 spermatozoa for each sample were counted. Percentages of viable spermatozoa were determined as the ratio: green cells/(green cells + red cells) × 100.

Sperm osmotic tolerance (SOT) was assessed using a hypo-osmotic swelling test (HOST) [14,15]. Five microliters of semen were added to 80 µL of distilled H_2_O and then stained with SYBR-14/PI and read as described above for sperm viability. 

This test is effective for assessing the percentage of viable spermatozoa that are capable of withstanding hypo-osmotic stress in vitro. Under hypo-osmotic conditions, viable thawed spermatozoa with intact membranes fluoresce green (SYBR) and exclude PI. Conversely, damaged membranes permit the passage of PI, staining spermatozoa that have lost their functional integrity red.

### 2.5. NMR Measurements

#### 2.5.1. Sample Preparation 

The Bligh–Dyer [51] method was used to extract and separate water-soluble and liposoluble metabolites from semen samples following the procedure previously reported [52].

Before NMR analysis, fresh and frozen semen was diluted at the same concentration and then centrifuged at 1500 rpm for 15 min to remove both diluent and seminal plasma. A chloroform/methanol (2:1, *v*/*v*) mixture was added to the sperm pellets, and the samples were homogenized with a vortex mixer for 60 s before adding distilled water in the proportion of 1:18:4 (spermatozoa–chloroform/methanol–water). The homogenate was centrifuged at a speed of 4000 rpm for 20 min at 5 °C. The liquid chloroform and water/methanol phases were separated and dried under vacuum in a rotary evaporator. The dried residues were dissolved into 0.75 mL of CDCl_3_/CD_3_OD (2:3 *v*/*v*) or 0.75 mL of D_2_O phosphate buffer (400 mM, pD = 7).

#### 2.5.2. NMR Spectra

The NMR spectra of aqueous and organic extracts were recorded at 27 °C on a Bruker AVANCE 600 NMR spectrometer operating at the proton frequency of 600.13 MHz and equipped with a Bruker multinuclear z-gradient inverse probe head capable of producing gradients in the z-direction with a strength of 55 G/cm. ^1^H spectra were referenced to methyl group signals of 3-(trimethylsilyl)-propionic-2,2,3,3-d4 acid sodium salt (TSP, δ = 0.00 ppm) in D_2_O and to the residual CHD_2_ signal of methanol (set to 3.31 ppm) in CDCl_3_/CD_3_OD mixture [53]. ^1^H spectra of aqueous extracts were acquired by coadding 512 transients with a recycle delay of 3 s. The residual HDO signal was suppressed using a standard Bruker presaturation sequence zgpr. The experiment was carried out by using a 45° pulse of 7.25 μs and 32,000 data points. ^1^H spectra of CDCl_3_/CD_3_OD extracts were obtained using the following parameters: 256 transients, 32,000 data points, a recycle delay of 3 s, and a 90° pulse of 10 μs. The ^1^H spectra were Fourier transformed using an exponential multiplication function with a line broadening factor of 0.3 Hz, and manual phase correction and baseline correction were applied.

#### 2.5.3. Measurement of the Metabolic Content in Aqueous Extract

The intensity of 21 ^1^H resonances due to water-soluble assigned metabolites (see Table 1) was measured with respect to the intensity of a TSP signal used as internal standard and normalized to 100.

#### 2.5.4. Measurement of the Metabolic Content in Organic Extracts

The integrals of 8 ^1^H resonances due to assigned liposoluble metabolites were measured and used to obtain the normalized integrals, see Table 1. All the integrals were normalized with respect to the integrals of α-CH_2_ groups of all fatty acid chains at 2.31 ppm set to 100%. The molar percentages of lipids were calculated taking into account the number of equivalent protons corresponding to a specific resonance. The resonances due to the CH_3_ of cholesterol (0.74 ppm), all allylic protons (2.08 ppm), α-CH_2_ groups of all fatty acid (2.31 ppm), CH_2_ diallylic protons of DUFA, (2.81 ppm), CH_2_ diallylic protons of PUFA (2.88 ppm), CH_2_N of PE (3.21 ppm), (CH_3_)_3_N^+^ of PC (3.28 ppm), and CH (double bond) proton of SMN (5.76 ppm) were integrated. The molar percentage of all saturated fatty chains (SFA) was calculated as 100 UFA, where UFA was calculated using the all-allylic-protons signal at 2.08 ppm.

### 2.6. Statistical Analysis 

Sperm qualitative parameters and metabolite levels determined by NMR analysis measured in fresh and frozen–thawed sperm were compared by paired-samples *t*-test (threshold at *p* < 0.05). Correlations between sperm variables and NMR-identified metabolites were assessed through Pearson’s correlation coefficients, setting significance thresholds at the *p* < 0.05 level (one-tailed) and *p* < 0.01 levels (two-tailed). All statistical tests were performed using the software package SPSS v23.0 (SPSS, Chicago, IL, USA).

## 3. Results

### 3.1. Sperm Quality

**Figure 1:**
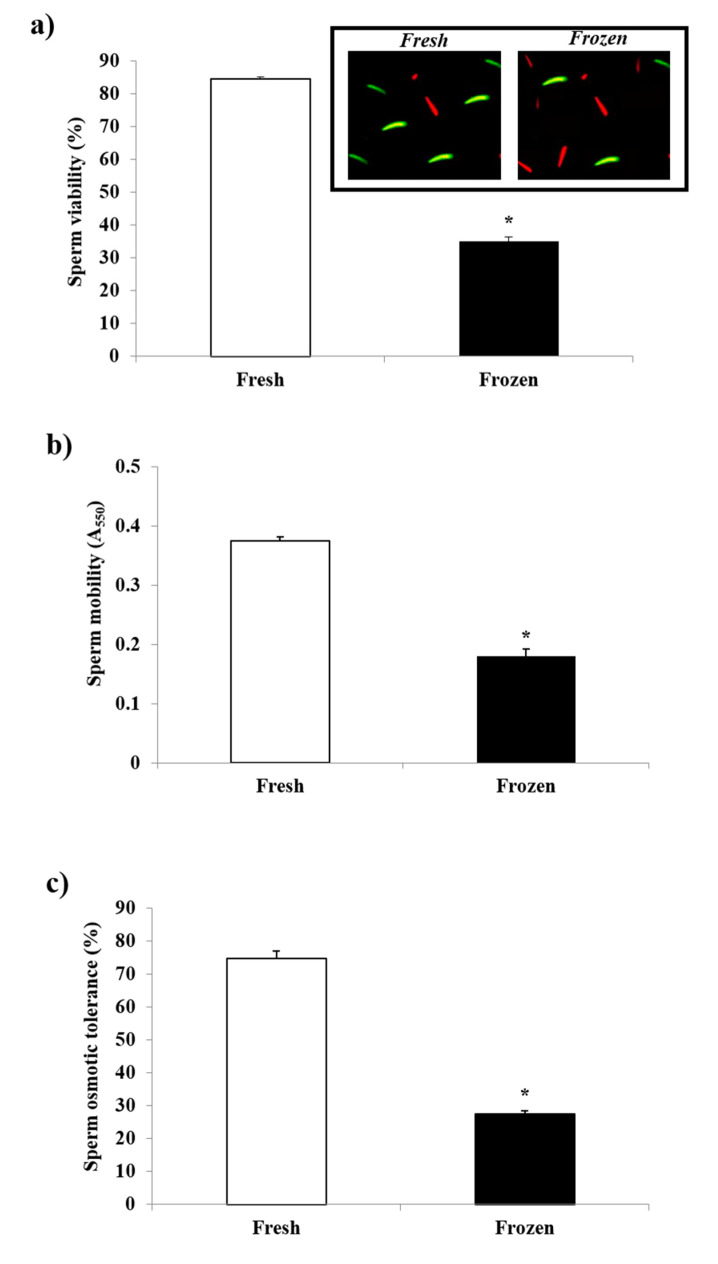
Effect of the cryopreservation process on the (**a**) viability, (**b**) mobility, and (**c**) osmotic tolerance of turkey sperm. Mean values ± SE (*n* = 5) of sperm qualitative parameters recorded for either fresh or frozen–thawed turkey sperm were reported. (**a**) Viability values, expressed as %, were measured by means of a dual staining technique (as shown in inset) using the stains SYBR-14 (green, viable cells) and PI (red, dead cells). (**b**) Mobility values, expressed as Abs 550 nm, were measured by the sperm motility test. (**c**) Sperm osmotic tolerance (SOT) was assessed by the hypo-osmotic H_2_O test. For further details, see Methods section. * = *p* < 0.05.

The sperm quality parameters recorded in freshly collected and frozen–thawed sperm samples are provided in Figure 1. The cryopreservation process severely affected all of the measured qualitative parameters. A significant reduction was found in sperm viability, as assessed by fluorescence microscopy (inset Figure 1a); these values, in fact, were lower by about 50% in frozen–thawed than in fresh samples (Figure 1a). A similarly remarkable decrease was observed in the sperm mobility of cryopreserved sperm (Figure 1b). The sperm osmotic tolerance also suffered from a dramatic decrease, with the values measured in cryopreserved sperm 40% lower than those measured in fresh samples (Figure 1c).

### 3.2. NMR Analysis

To obtain a picture of the metabolite changes due to cryopreservation, water and lipid soluble components in turkey sperm were identified in NMR spectra using the same NMR experimental conditions and assignments from our previous study [49]. All identified metabolites quantified for both fresh and cryopreserved samples are reported in Table 1. The identified water-soluble metabolites were the amino acids alanine (Ala), isoleucine (Ile), phenylalanine (Phe), leucine (Leu), valine (Val), aspartic acid (Asp), glycine (Gly), tyrosine (Tyr), glutamate (Glu) and glutamine (Gln) and other water-soluble metabolites such as lactate, acetate, citrate, creatine, acetyl-carnitine (ac-carnitine), carnitine, glucose, *myo*-inositol, fumaric acid, formic acid, and adenosine monophosphate (AMP).

Compared with fresh samples, frozen–thawed sperm showed decreased content of Ala, Ile, Leu, Tyr, Val (*p* < 0.01), and Phe (*p* < 0.05), whereas Gly levels proved to increase in cryopreserved samples. No statistically significant differences were found for Asp, Glu, or Gln. 

Moreover, significant decreases (*p* < 0.01) in formate, lactate, AMP, carnitine, and creatine levels were found in frozen–thawed sperm, which also showed increased values of citrate (*p* < 0.05) and ac-carnitine (*p* < 0.01). No significant differences were observed between fresh and frozen sperm for acetate, fumarate, glucose, or *myo*-inositol content.

In lipid extract, cholesterol (CHO), the total content of all unsaturated fatty acids (UFA), diunsaturated fatty acids (DUFA), phosphatidylcholine (PC), phosphatidylethanolamine (PE), sphingomyelin (SMN), polyunsaturated fatty acids (PUFA), and the total content of saturated fatty acids (SFA) were identified.

Significantly higher values of CHO, PUFA (*p* < 0.05), and UFA (0.01) were recorded in fresh than in frozen–thawed sperm, whereas a higher value of SFA was observed in the latter samples (*p* < 0.01). No significant differences between fresh and frozen–thawed samples were scored for other lipids identified. 

### 3.3. Correlation

In order to study the correlations between sperm quality parameters and the amounts of the different metabolites, Pearson correlation coefficients were calculated (Table 2).

Sperm mobility, viability, and osmotic tolerance were positively correlated with Ala, Ile, Leu, Phe, Tyr, Val, formate, lactate, carnitine (*p* < 0.01), AMP, CHO, and PC (*p* < 0.05). The content of creatine was found to be positively correlated with sperm vitality and osmotic tolerance (*p* < 0.05). UFA levels correlated positively with sperm motility and osmotic tolerance (*p* < 0.05), and PE content showed positive correlations with sperm motility and sperm viability (*p* < 0.05). On the contrary, negative correlations with all qualitative parameters were found for Gly, citrate (*p* < 0.01), and ac-carnitine (*p* < 0.05), and both fumarate and SFA (*p* < 0.05) were negatively correlated with sperm motility and sperm osmotic tolerance.

## 4. Discussion

Results obtained by sperm quality analysis showed that the freezing–thawing process caused significant reductions in sperm motility, viability, and osmotic tolerance. These findings were in accordance with previous reports on avian sperm [14,15,18,54]. In particular, the post-thaw semen quality measured in this study was similar to that observed in a previous paper [14]: after thawing, in fact, the returned recovery rates (value found in cryopreserved semen/value found in the fresh semen × 100) were about 40%, 48%, and 37% for sperm viability, mobility, and osmotic tolerance, respectively. 

It is well known that the semen cryopreservation process imposes numerous stresses not only on the physical features of sperm but on its chemical composition, which in turn is essential for sperm function, as in the case of energy metabolism, which is known to be absolutely crucial in supporting sperm motility. Thus, the high sensitivity of turkey sperm to the cryopreservation process is assumed to be a consequence of the sperm metabolic profile of this bird (also see [10]). In particular, the first pieces of evidence about changes in lipid content, cholesterol/phospholipid ratios, and glycoconjugate and ATP content as a result of semen cryopreservation process have been already reported [18,55,56]. However, a more exhaustive picture of the sperm metabolic profile before and after cryopreservation is still lacking. Therefore, in this study, by taking advantage of NMR technique, a relevant number of metabolites were assessed in both fresh and frozen–thawed spermatozoa in order to correlate their levels to sperm quality variations.

Data obtained by NMR analysis are discussed separately for each class of compounds.

*Amino Acids*. We observed a general decrease in amino acid levels in frozen–thawed sperm, with statistically significant differences for Ala, Ile, Leu, Phe, Tyr, and Val. Hence, we hypothesize that the reduction in the levels of these amino acids could play a key role in the reduced quality of post-thaw sperm. This notion is substantiated by the positive correlations detected here between sperm viability, mobility, and osmotic tolerance and these amino acids. Thus, it could be hypothesized that increasing the sperm content of these amino acids could improve the freezability of turkey semen and, consequently, the post-thaw sperm quality. This is also consistent with previous research involving both human [40] and other mammalian species [57,58,59,60,61,62] in which the supplementation of amino acids was successfully used to improve post-thawing sperm quality. More recently, amino acid supplementation was successfully checked in chicken [63,64,65,66].

To date, the mechanism by which amino acids could provide cryoprotection is not fully understood and remains unclear [60]. However, some authors have proposed that amino acids could form a layer over the sperm surface via the electrostatic interaction with the phosphate group of the sperm plasma membrane phospholipids, acting as a cushion for damage against ice crystal formation and therefore preventing thermal shock [57]. Thus, amino acids could also interact with phospholipid bilayers during freezing, allowing stabilization of the cell membrane [2]. In addition, the supplementation of amino acids to semen diluents can lead to a reduction in the concentration of toxic solutes to levels associated with lesser toxicity; moreover, some amino acids can protect sperm cells against the denaturing effects of hyperosmolality during cryopreservation process [2,67]. In accordance with our results, in a recent study, the supplementation of valine to chicken freezing extenders resulted in a decrease in DNA fragmentation and a positive effect on the fertilizing ability of frozen–thawed sperm, with a better response in a breed that is considered to have the lowest semen freezability [64].

In addition to the cryoprotective role played by exogenous amino acids as extender supplementation, putative effects of endogenous amino acids in specific sperm function should be taken in consideration. It was shown, in fact, that seminal plasma levels of Leu were higher in high- than in low-fertility bulls [68]; this was proposed to be due to the action of Leu in modulating active Ca^2+^ transport across sperm membrane, which would result in a delay in Ca^2+^ uptake in ejaculated sperm. Leu was also proposed to be one of the fertility biomarkers in bovine species [68]. Accordingly, free Leu content in chicken seminal plasma was positively correlated with sperm viability as well as DNA integrity [69].

Contrary to levels of the majority of detected amino acids, Gly levels increased in frozen–thawed samples, and a significant negative correlation between its levels and sperm osmotic tolerance (SOT) was found. This resembles what was already observed in a previous study, in which a decrease in SOT was associated with increased levels of Gly in sperm of ageing turkey males [49]. Surprisingly enough, Gly content was also found to be negatively correlated with sperm motility. This seems to be in contrast with other reports showing that the addition of Gly to the diluent prevented significant changes in chicken sperm motility during the freezing–thawing procedure [70]. However, this dissonant result could be due to differences in metabolism between the two avian species [71], as well as a different mechanism of action for Gly as a function of its concentration [70]. Nonetheless, further investigation into the role of amino acids in turkey sperm metabolism should be carried out in future studies. 

*Other water-soluble metabolites.* Similarly to what was observed for most amino acids, significant reductions in carnitine, lactate, formate, creatine, and AMP content were found in cryopreserved sperm.

Furthermore, positive correlations between the aforementioned metabolites and sperm quality parameters were found, suggesting that these metabolites could somehow be involved in the decrease in sperm quality occurring in cryopreservation.

It is not surprising that a reduced post-thawed semen quality could be related to low content of carnitine. It is known, in fact, that carnitine plays a key role in sperm metabolism by providing readily available energy, thus affecting sperm motility and maturation and the spermatogenic process [46]. This should be more evident in turkey sperm, which presents a highly oxidative metabolism [71,72]. Moreover, carnitine has a protective action against reactive oxygen species (ROS) by exerting antioxidant properties [73,74]. The results therein were consistent with previous studies reporting that the addition of L-carnitine in the extender enhanced chicken sperm motility in vitro during liquid storage and frozen state [2,66,75] and that supplementation of L-carnitine in the diet could improve drake semen quality [76]. Accordingly, L-carnitine supplementation to freezing extender improved human sperm motility and vitality and reduced sperm DNA oxidation during cryopreservation [77].

The reduction in free carnitine levels observed in frozen–thawed sperm could also be due to the slight, but significant, increase in ac-carnitine levels observed in these samples, which was correlated with a sperm quality decrease. Since ac-carnitine seems to be involved in buffering or trapping the excessive production of acetyl-CoA [45], it could be speculated that as a result of cryopreservation process, overproduction of acetyl-CoA could occur, which in turn could increase ac-carnitine levels in sperm. In agreement with this hypothesis, cryopreserved samples also showed an increase in citrate, which derives from the condensation between oxaloacetate and acetyl-CoA. Thus, in frozen–thawed sperm, the increases in the amounts of both ac-carnitine and citrate, together with the unvaried levels of other Krebs cycle compounds such as fumarate, may suggest a reduced capability of mitochondria to utilize acetyl-CoA through this pathway and/or a reduced capability of the mitochondrial respiratory chain (linked to the Krebs cycle by its products NADH and FADH_2_) to support sperm energy requirement. In this regard, it must be noted that among avian species, turkey presents a high oxidative metabolism [10]. The high aerobic metabolism for this species, in fact, was already reported [78] and further highlighted by a previous study in which it was found that the stimulation of the cytochrome c oxidase (complex IV of the respiratory chain) by He–Ne laser light increased post-thaw sperm motility in turkey, but not in chicken or pheasant [71].

In addition to mitochondrial oxidative phosphorylation, glycolysis constitutes the other energy source in sperm. In this regard, it was recently reported that water-soluble extract of quail cloacal gland secretion contained glucose as an energy source for the intrinsic sperm mobility after transportation to female vagina [79]. However, in partial agreement with the hypothesis of a cryopreservation-dependent reduction in mitochondrial activity, here we found no differences in the levels of glucose between fresh and cryopreserved samples. Thus, in our case, a reduced sperm cell capability of glucose uptake due to putative impairment of glucose carriers should be ruled out. Contrarily, in other species such as boars, it was found that the cryopreservation process impaired glucose uptake by affecting the distribution of glucose transporters, especially GLUT-3 [80], a GLUT family member that is also present in avians, as shown by proteomic and peptidomic analyses of chicken sperm [81]. On the other hand, the impairment of the glycolytic pathway as a result of cryopreservation also seems unlikely in light of the supposed increased acetyl-CoA production discussed above.

Despite unaltered glucose levels, in cryopreserved samples, a significant reduction in lactate content was found, which was correlated with the decreased sperm quality in these samples. This positive corelation is not surprising, since L-lactate was found to play an active role in sperm bioenergetics because of its mitochondrial metabolism [82,83]. In particular, because of the occurrence of a mitochondrial L-lactate dehydrogenase [84,85,86], mitochondria are able to actively metabolize this substrate for energy purposes [82]. For this reason, a reduction in sperm energy fuel such as L-lactate in cryopreserved sperm could be responsible for a decreased sperm quality. Therefore, further investigation of possible changes occurring in cryopreservation in cytosolic and mitochondrial L-lactate dehydrogenase (both protein levels and enzyme activities) represents a task to be addressed in future studies. 

*Myo*-inositol levels were also investigated, since it has been reported that its addition to sperm could improve sperm motility [87] and mitochondrial membrane potential [88] (also see [89]). However, as for glucose, no significant variation between fresh and frozen–thawed sample was found. Thus, *myo*-inositol should not play a role in the sperm quality decrease during turkey semen cryopreservation, at least in our experimental conditions. 

Another interesting result obtained by analyzing the water-soluble fraction of frozen–thawed sperm was the decreased AMP content in these samples. This low AMP content could also partially explain the observed increase in citrate levels, since the Krebs cycle rate is extremely sensitive to AMP concentration [90]. It is known, in fact, that AMP acts as a cell energy sensor via the AMP/ATP ratio; its increase is a signal of cell energy deficiency and leads to activation of the AMP-activated protein kinase (AMPK) [91], a mechanism well reported also in avian sperm [92]. Thus, the observed reduction in the levels of AMP in cryopreserved samples is quite surprising, especially in light of the 40-fold decrease in ATP levels that was already found as a result of the freezing–thawing process of turkey sperm [56]. In this regard, it must be noted that cell levels of AMP, ADP, and ATP give rise to the adenylate energy charge; moreover, these compounds are closely related to each other by the activity of the enzyme adenylate kinase (AK), which catalyzes the following equilibrium reaction: 2ADP = ATP + AMP. The activity of this enzyme was reported to allow ADP to partially support sperm motility [93], at least under energetic stress conditions [94]. Since this scenario seems to occur in turkey sperm cryopreservation, as evidenced by the dramatic ATP decrease, it could be speculated that a reduction in AK activity could play a role in the sperm quality decrease. Thus, further investigation into this aspect (i.e., the occurrence and activity of AK in turkey sperm and its variation during the freezing–thawing procedure), as well as the detection of ADP levels in sperm before and after cryopreservation, appears to be mandatory in further studies.

*Lipids.* It is widely accepted that lipids are the main component of sperm membrane and that they are responsible for the fluidity of membrane bilayers [95], which in turn influences the spermatozoa freezability of different animal species [48,96]. Accordingly, one study showed that the freezing–thawing process resulted in a rigidifying effect on the sperm membrane and suggested that sperm adaptability to freezing–thawing-induced stress could be dependent on its initial membrane fluidity [97]. The same authors argued that the initial membrane fluidity had practical implications for predicting the response of spermatozoa following freezing and thawing and for improving the recovery of viable spermatozoa.

Thus, by turning our attention to lipid extract, in this study, we observed significant reductions in CHO, UFA, PUFA and an increase in SFA in frozen compared with fresh sperm. In addition, cholesterol was positively correlated with sperm viability, motility, and osmotic tolerance. This outcome leads us to assume that the reduction in sperm quality after freezing–thawing could be due to increased spermatozoon membrane rigidity accompanied by the decrease in the cholesterol/phospholipid ratio. This would be consistent with previous studies in human [97] and in turkey [18,98] reporting that semen storage and, more importantly, the freezing–thawing procedure induced a rigidifying process in the sperm membrane followed by a dramatic decrease in the cholesterol/phospholipid ratio.

Moreover, besides the cholesterol content, membrane fluidity depends on the degree of saturation of fatty acids in membrane phospholipids [99], since saturated fatty acids are rigidifying components of the mammalian sperm membranes. The avian sperm membrane contains more polyunsaturated fatty acids (PUFAs) than mammal sperm and has a lower protein content, a lower cholesterol/phospholipid ratio, and greater overall fluidity at physiological temperatures [2]. Here, we found an increase in the SFA/UFA ratio occurring in cryopreservation, since it was 0.62 and 0.75 in fresh and frozen–thawed sperm, respectively. Thus, we can conclude that, distinctly from semen liquid storage, in which SFA/UFA ratio was not affected [100], more drastic storage conditions such as cryopreservation strongly affect the lipid composition, and consequently the fluidity, of turkey sperm membrane. In this regard, previous studies showed that the lipid composition of avian spermatozoa could be modified by the diet with subsequent effects on membrane fluidity [22,32,101]. Thus, a diet inducing a lower SFA/UFA ratio in turkey sperm could be tried out in the future as a putative way to increase the freezability of avian sperm. At the same time, it must be taken in consideration that sperm cryopreservation strongly increases ROS production [102,103], resulting in lipid peroxidation. Therefore, the reduction in sperm quality could also be due to a membrane destabilization deriving from PUFA peroxidation [98]. Thus, a higher UFA content in the sperm membrane may not necessarily be the best way to improve sperm freezability. However, proper knowledge of the relationship between metabolic profile and the freezability of the spermatozoa remains very interesting in light of the possibility of modifying the sperm metabolic profile via various factors such as diet manipulation, strain, and ageing [22,32,33,49].

## 5. Conclusions

We are confident that the findings reported here provide a valid contribution to the scientific community, since they returned a clearer scenario of metabolic changes occurring in turkey sperm cryopreservation. Semen cryopreservation is an important biotechnological strategy used to both preserve and protect genetic resources, which are subject to increasingly serious reductions in some species, as well as to enhance animal biodiversity in the case of inbreeding risks. The knowledge of metabolites responsible for the post-thawing sperm quality decrease allows (*i)* identifying several biological markers related to the sperm freezability of male turkey donators and (*ii)* suggesting a supplementation of specific metabolites in the diet or in the freezing medium in order to obtain spermatozoa abler to withstand the freezing process.

In addition, the analytic approach used here, which resorted to the NMR technique to determine the metabolites involved in semen cryopreservation, constitutes an important tool that can be applied also to other species, including humans.

## Figures and Tables

**Table 1:** Metabolites identified and quantified by NMR in fresh and frozen sperm of turkey males at 32 weeks of age.

Metabolite, ^1^H Chemical Shift (ppm)	Fresh	Frozen	
	Water Extract		*p-Value*
*Amino acids*	mol % (*n* = 5)	mol % (*n* = 5)	
**Ala (1.48)**	**0.178 ± 0.007 ^a^**	**0.113 ± 0.007 ^b^**	***0.001***
Asp (2.83)	0.239 ± 0.022 ^a^	0.221 ± 0.005 ^a^	*0.466*
Gln (2.45)	1.614 ± 0.314 ^a^	1.468 ± 0.197 ^a^	*0.412*
Glu (2.07)	66.547 ± 0.391 ^a^	66.956 ± 0.761 ^a^	*0.725*
**Gly (3.57)**	**4.648 ± 0.043 ^a^**	**4.951 ± 0.073 ^b^**	***0.001***
**Ile (1.02)**	**0.015 ± 0.001 ^b^**	**0.009 ± 0.001 ^b^**	***0.009***
**Leu (0.96)**	**0.054 ± 0.003 ^a^**	**0.026 ± 0.001 ^b^**	***0.003***
**Phe (7.43)**	**0.022 ± 0.001 ^a^**	**0.017 ± 0.001 ^b^**	***0.030***
**Tyr (6.92)**	**0.048 ± 0.002 ^a^**	**0.020 ± 0.002 ^b^**	***0.001***
**Val (0.99)**	**0.038 ± 0.002 ^a^**	**0.019 ± 0.002 ^b^**	***0.002***
***Organic acids***			
Acetate (1.93)	0.486 ± 0.064 ^a^	0.353 ± 0.087 ^a^	*0.403*
**Citrate (2.57)**	**0.089 ± 0.006 ^b^**	**0.125 ± 0.006 ^a^**	***0.039***
**Formate (8.46)**	**0.039 ± 0.003 ^a^**	**0.019 ± 0.004 ^b^**	***0.002***
Fumarate (6.53)	0.032 ± 0.003 ^a^	0.040 ± 0.002 ^a^	*0.072*
**Lactate (1.33)**	**1.362 ± 0.072 ^a^**	**0.884 ± 0.059 ^b^**	***0.001***
***Other compounds***			
**Ac-carnitine (3.20)**	**0.037 ± 0.002 ^a^**	**0.045 ± 0.002 ^b^**	***0.006***
**AMP (8.28)**	**0.146 ± 0.007 ^a^**	**0.115 ± 0.008 ^b^**	***0.005***
**Carnitine (3.24)**	**0.080 ± 0.005 ^a^**	**0.037 ± 0.002 ^b^**	***0.002***
**Creatine (3.94)**	**1.828 ± 0.136 ^a^**	**1.431 ± 0.071 ^b^**	***0.028***
Glucose (3.26 and 5.25)^*^	16.445 ± 0.437 ^a^	17.085 ± 0.592 ^a^	*0.504*
*Myo*-inositol (3.65)	6.054 ± 0.039 ^a^	6.067 ± 0.205 ^a^	*0.949*
	**Lipid extract**		
	**mol % (*n* = 3)**	**mol % (*n* = 3)**	
**CHO (0.74)**	**9.587 ± 0.348 ^a^**	**6.944 ± 0.533 ^b^**	***0.036***
**SFA**	**38.001 ± 1.430 ^b^**	**43.088 ± 1.032 ^a^**	***0.006***
DUFA (2.81)	4.200 ± 0.136 ^a^	3.857 ± 0.356 ^a^	*0.293*
**UFA (2.08)**	**62.000 ± 1.430 ^a^**	**56.912 ± 1.032 ^b^**	***0.006***
**PUFA (2.86)**	**36.793 ± 0.561 ^a^**	**34.665 ± 0.633 ^b^**	***0.027***
PC (3.28)	24.703 ± 0.760 ^a^	19.667 ± 0.612 ^a^	*0.081*
PE (3.21)	14.107 ± 0.152 ^a^	11.989 ± 0.611 ^a^	*0.072*
SMN (5.76)	6.990 ± 0.239 ^a^	6.200 ± 0.577 ^a^	*0.167*

^a,b^ Different superscript letters within the same row indicate significant differences (*p* < 0.05). Abbreviations:Ac-carnitine: acylcarnitine; AMP: adenosine monophosphate; CHO: cholesterol; SFA: total content of saturated fatty acids; DUFA: diunsaturated fatty acids; UFA: total content of unsaturated fatty acids; PUFA: polyunsaturated fatty acid; PC: phosphatidylcholine; PE: phosphatidylethanolamine; SMN: sphingomyelin.

**Table 2:** Pearson correlations between sperm qualitative parameters and metabolites identified in fresh and frozen–thawed sperm of turkey males at 32 weeks of age.

Metabolite	Sperm Variables		
	Mobility	Viability	Osmotic Tolerance
Ala	0.867 **	0.930 **	0.902 **
Gly	−0.770 **	−0.771	−0.802 **
Ile	0.818 **	0.861 **	0.908 **
Leu	0.915 **	0.942 **	0.962 **
Phe	0.683 *	0.784 **	0.781 **
Tyr	0.915 **	0.969 **	0.972 **
Val	0.871 **	0.937 **	0.947 **
Citrate	−0.874 **	−0.833 **	−0.815 **
Formate	0.764 *	0.854 **	0.811 **
Fumarate	−0.723 *		−0.659 *
Lactate	0.806 **	0.887 **	0.830 **
Ac-carnitine	−0.740 *	−0.695 *	−0.689 *
AMP	0.653 *	0.728 *	0.708 *
Carnitine	0.923 **	0.925 **	0.957 **
Creatine		0.673 *	0.697 *
CHO	0.907 *	0.884 *	0.876 *
SFA	−0.865 *		−0.868 *
UFA	0.865 *		0.868 *
PC	0.969 **	0.882 *	0.884 *
PE	0.851 *	0.870 *	

Pearson correlation coefficients were calculated for the sperm qualitative parameters (Figure 1) versus metabolites detected by NMR (Table 1). Only significant correlation values * at the 0.05 level and ** at the 0.01 level are reported. Abbreviations:Ac-carnitine: acylcarnitine; AMP: adenosine monophosphate; CHO: cholesterol; SFA: total content of saturated fatty acids; UFA: total content of unsaturated fatty acids; PC: phosphatidylcholine; PE: phosphatidylethanolamine.

## Data Availability

The datasets used are available from the corresponding author on reasonable request.
